# Supplementary material for: Transcriptional analysis reveals that the intracellular lipid accumulation impairs gene expression profiles involved in insulin response-associated cardiac functionality
Source: Sci Rep. 2023 May 30;13:8761. doi: 10.1038/s41598-023-35951-6 (PMC10229538; doi:10.1038/s41598-023-35951-6)
Supplement: Supplementary file 1 — Supplementary Figures. [file 41598_2023_35951_MOESM1_ESM.pdf]

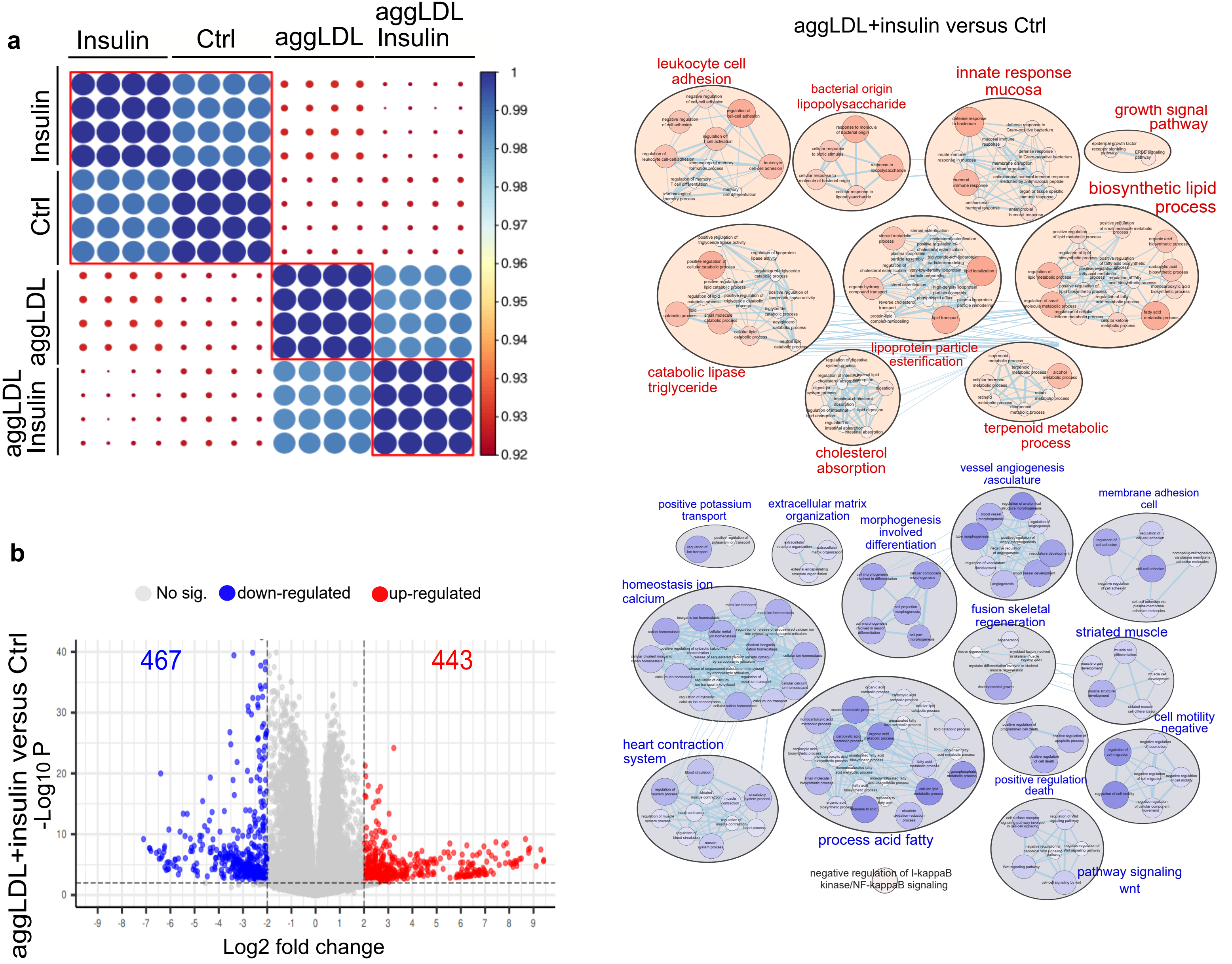

Supplementary Figure 1. Cells were stimulated or not (Ctrl) with aggLDL 100 µg/mL for 8 h and then with insulin 100 nM for 2h. (a) Expression matrix-based Pearson's correlation analysis. (b) Volcano plot graph shows differentially expressed genes (DEG) for |FC|≥2; p-value<0.01; and FDR<0.05. aggLDL + insulin condition was compared with the control. The number of up- and downregulated genes is shown in the graph. (c) Gene Ontology (GO) (Biological Process) enrichment analysis. Red nodes indicate upregulated processes and blue ones downregulated. The size and intensity of the nodes' colours are proportional to the number of genes enriching this biological process. The lines connecting the nodes represent the biological processes related to each other.

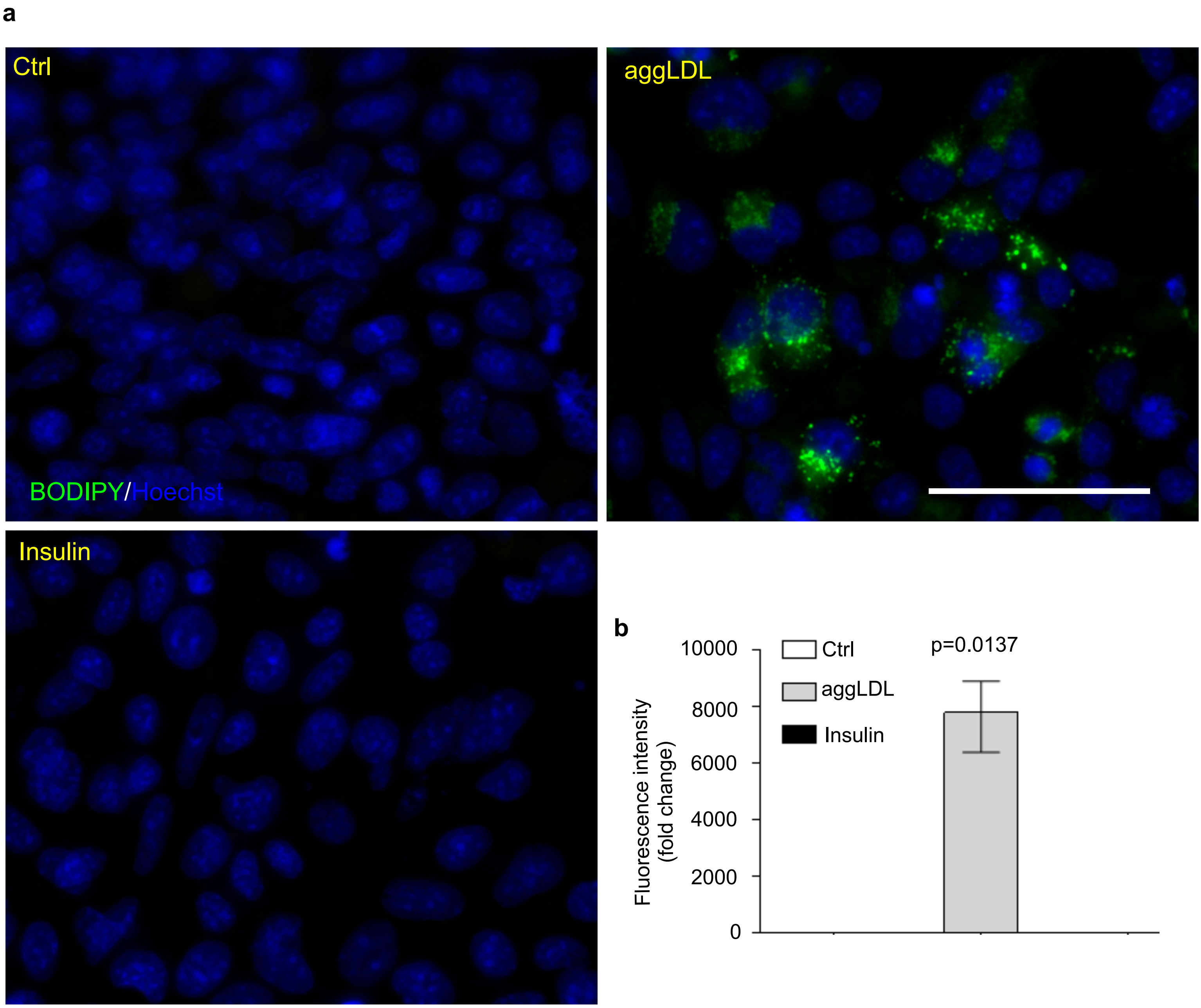

Supplementary Figure 2. Neutral lipid accumulation in HK-1 cardiomyocytes (a) Fluorescence microscopy images showing BODIPY-stained lipid droplets (green) in HL-1 cardiomyocytes treated with 100 µg/mL aggLDL for 8 h or insulin 100 nM for 2 h. Cell nuclei are stained with Hoechst (blue). Scale bar: 15 µm. (n=20 per condition). (b) Graph represents mean ± SEM of the fluorescence intensity of BODIPY per cell area expressed as a fold change compared to the mean of control condition. * $p<0.05$ are considered significant.

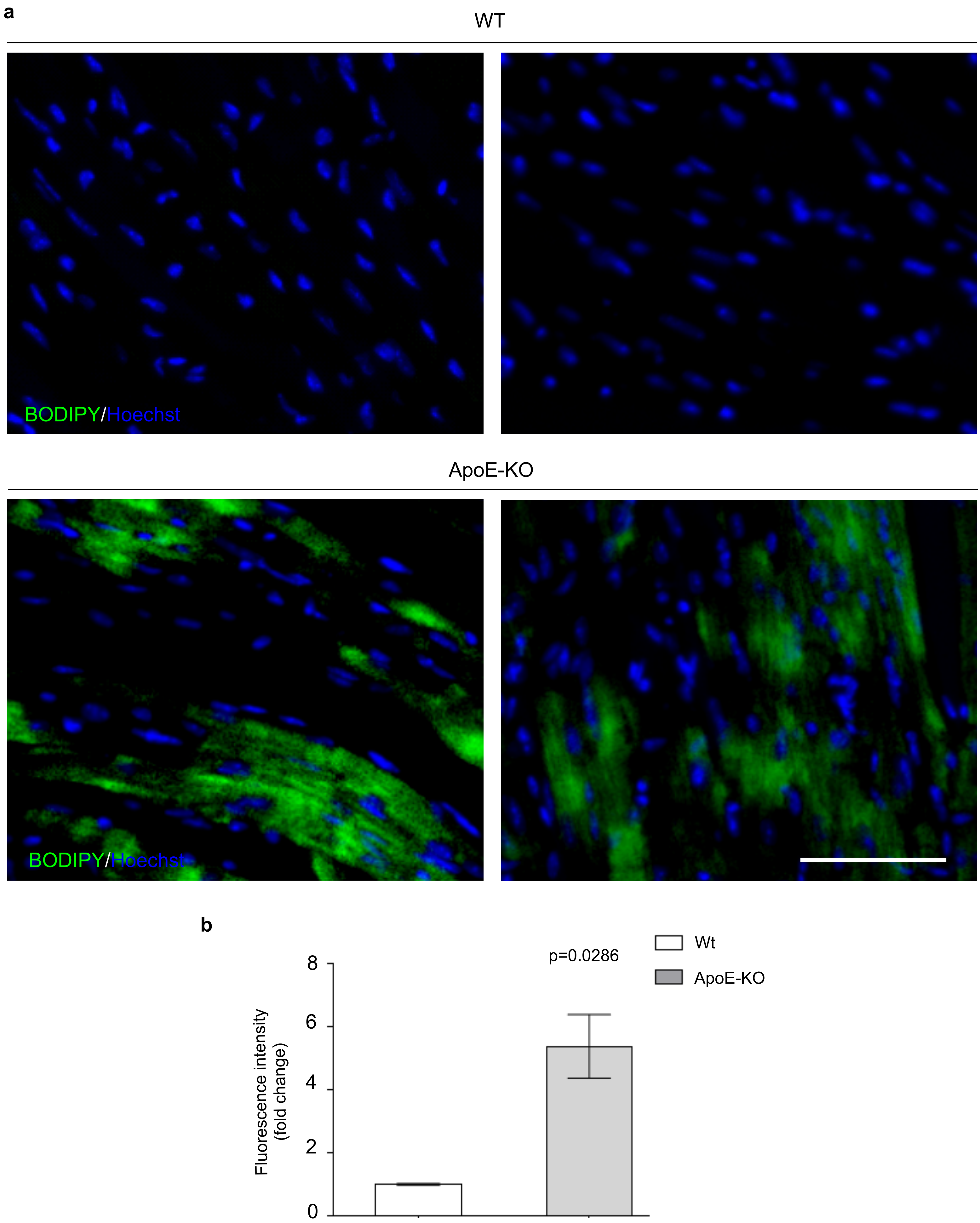

Supplementary Figure 3. Neutral lipid accumulation in myocardium of ApoE-KO mice (a) Fluorescence microscopy images showing BODIPY-stained lipid droplets (green) in cryosections of hearts from ApoE-KO or Wt mice (6 months of age). Cell nuclei are stained with Hoechst (blue). Scale bar: 50 μm. (n=9/group). (b) Graph represents mean ± SEM of the fluorescence intensity of BODIPY per tissue area expressed as a fold change.
